# Soluble fms-Like Tyrosine Kinase 1 (sFlt1), Endoglin and Placental Growth Factor (PlGF) in Preeclampsia among High Risk Pregnancies

**DOI:** 10.1371/journal.pone.0013263

**Published:** 2010-10-11

**Authors:** Robert W. Powers, Arun Jeyabalan, Rebecca G. Clifton, Peter Van Dorsten, John C. Hauth, Mark A. Klebanoff, Marshall D. Lindheimer, Baha Sibai, Mark Landon, Menachem Miodovnik, for the Eunice Kennedy Shriver National Institute of Child Health Human Development Maternal-Fetal Medicine Units Network

**Affiliations:** 1 Department of Obstetrics, Gynecology and Women's Health, University of Pittsburgh School of Medicine, Pittsburgh, Pennsylvania, United States of America; 2 The George Washington University Biostatistics Center, Washington, D.C., United States of America; 3 Department of Obstetrics and Gynecology, Medical University of South Carolina, Charleston, South Carolina, United States of America; 4 Department of Obstetrics and Gynecology, University of Alabama at Birmingham School of Medicine, Birmingham, Alabama, United States of America; 5 Eunice Kennedy Shriver National Institute of Child Health and Human Development, Bethesda, Maryland, United States of America; 6 Department of Obstetrics and Gynecology, University of Chicago School of Medicine, Chicago, Illinois, United States of America; 7 Department of Obstetrics and Gynecology, University of Tennessee School of Medicine, Memphis, Tennessee, United States of America; 8 Department of Obstetrics and Gynecology, The Ohio State University School of Medicine, Columbus, Ohio, United States of America; 9 Department of Obstetrics and Gynecology, University of Cincinnati School of Medicine, Cincinnati, Ohio, United States of America; Institute of Zoology, Chinese Academy of Sciences, China

## Abstract

**Background:**

Differences in circulating concentrations of antiangiogenic factors sFlt1 and soluble endoglin (sEng) and the pro-angiogenic growth factor PlGF are reported to precede the onset of preeclampsia weeks to months in low-risk pregnant women. The objective of this study was to investigate whether similar changes can be detected in pregnant women at high-risk to develop the syndrome.

**Methods:**

This study is a secondary analysis of the NICHD MFMU trial of aspirin to prevent preeclampsia in high-risk pregnancies. Serum samples were available from 194 women with pre-existing diabetes, 313 with chronic hypertension, 234 with multifetal gestation, and 252 with a history of preeclampsia in a previous pregnancy. Samples collected across pregnancy were analyzed in a blinded fashion for sFlt1, sEng and PlGF.

**Results:**

The odds of developing preeclampsia were significantly increased among women with multiple fetuses for each 2-fold elevation in sFlt1, sEng and the ratio of angiogenic factors (e.g. OR 2.18, 95% CI 1.46-3.32), and significantly decreased for each 2-fold elevation in circulating PlGF (OR 0.50, 95% CI 0.30-0.82) between 7 and 26 weeks' gestation. Cross-sectional analysis of the angiogenic factors across gestation showed significant differences during the third trimester in women who develop preeclampsia compared with appropriate controls in all high-risk groups. However, when data were examined in relation to the gestational week when preeclampsia was diagnosed only sFlt1 was significantly higher 2 to 5 weeks before the clinical onset of preeclampsia and only in women with previous preeclampsia.

**Conclusions:**

The pattern of elevated concentrations of sFlt1 and sEng, and low PlGF in high-risk pregnant subjects who develop preeclampsia is similar to that reported in low-risk pregnant women. However, differences in these factors among high-risk women who do and do not develop preeclampsia are modest, and do not appear to be clinically useful predictors in these high-risk pregnant women.

## Introduction

Preeclampsia is a pregnancy-specific syndrome affecting approximately 5% of all pregnancies and is a leading cause of maternal and fetal morbidity and mortality worldwide [1], [2], [3]. The incidence of preeclampsia is 3-5x greater in women with chronic hypertension, diabetes present before conception, multifetal gestation, and women who had preeclampsia in a previous pregnancy, and these populations are labeled “high-risk” [4].

The pathogenesis of preeclampsia remains incompletely elucidated, however increased attention has been directed toward the role of angiogenic and antiangiogenic factors including elevated soluble fms-like tyrosine kinase 1 (sFlt1) and soluble endoglin (sEng, a receptor for members of the TGFβ superfamily), and lower placental growth factor (PlGF) [5], [6], [7], [8]. Concentrations of these factors are significantly different in low-risk women who later develop preeclampsia several weeks to months before clinical manifestations of the disorder when compared with similar low-risk women who have uncomplicated pregnancies [6], [9]. However, there is limited information regarding changes in these circulating factors during pregnancies of high-risk patients who later develop preeclampsia compared with similarly at risk women who do not develop preeclampsia, and the ability of measured levels of sFlt1, sEng and PlGF to predict preeclampsia in these high-risk patients.

The goal of this study was to investigate whether differences in the angiogenic factors sFlt1, sEng and PlGF in high-risk patients would identify women who later develop preeclampsia in a manner similar to reports in the literature regarding low-risk women. The high-risk groups studied in this secondary analysis, chronic hypertensive, pre-pregnancy diabetes, women with multifetal gestation, or previous preeclampsia, were analyzed separately following a pre-analysis hypothesis that the pathogenesis of preeclampsia for each group may be different. This also permitted investigation of possible differences in angiogenic factor levels between the groups.

## Methods

### Study Population

We performed a secondary analysis of serum samples obtained during the Maternal-Fetal Medicine Units Network multicenter randomized controlled trial of low-dose aspirin for the prevention of preeclampsia in high-risk women [4]. This study, conducted between May 1991 and June 1995, investigated four groups of high-risk women: those with pre-gestational insulin treated diabetes mellitus, chronic hypertension, multifetal gestation, and those with a history of preeclampsia in a previous pregnancy. At enrollment, subjects were offered participation in an ancillary study that collected blood samples at three time points (study entry, 24–28 and 34–38 weeks' gestation). The ancillary study started after the original trial was in progress and only a subset of trial subjects participated. Subjects were included if they had at least a study entry sample available for analysis.

The diagnosis of chronic hypertension required documentation of antihypertensive-drug therapy by medical records or a blood pressure while sitting of greater than or equal to 140/90 mmHg taken on two occasions at least four hours apart, either before pregnancy or during pregnancy, but prior to study entry and before 20 weeks' gestation. Multifetal gestation was documented by ultrasound examination before enrollment. Previous preeclampsia was defined as new-onset proteinuric hypertension as determined by medical records or, in the absence of a record, an oral history of preeclampsia that resulted in delivery before the 37^th^ gestational week. At the screening visit, all women underwent urinary-protein testing by dipstick. If the test was 1+ or greater, a 24-hour urine sample was collected; women with values of 300mg of protein per 24 hours were considered to have proteinuria. Women with multi-fetal gestations were ineligible for the study if they also had diabetes mellitus, chronic hypertension, or proteinuria as defined above, as were women with a history of preeclampsia and current proteinuria. Women with both diabetes and hypertension were included in the diabetes group.

Institutional Review Board approval for the use of these stored samples was obtained at both the University of Pittsburgh and George Washington University. Subjects provided written informed consent prior to enrolling in the initial study. Laboratory assessment was completed at Magee-Womens Research Institute, and data was subsequently linked and statistical assessment completed at the George Washington University Biostatistics Center.

### Diagnosis of Preeclampsia

The diagnosis of preeclampsia for each of these high-risk groups has been described previously [4]. In brief, preeclampsia was defined in normotensive women with normal urinary protein excretion at baseline as the development of hypertension plus one of the following: proteinuria, thrombocytopenia, or pulmonary edema. Hypertension was defined as a systolic and/or diastolic blood pressure equal to or greater than 140 mmHg and 90 mmHg respectively, on two occasions at least four hours apart and within the same period (antepartum, intrapartum, postpartum). Proteinuria was defined as excretion of ≥ 300mg of protein in a 24-hour urine collection, or two dipstick-test results of ≥ 2+ (100 mg per deciliter), the values recorded at least 4 hours apart, with no evidence of urinary tract infection. Thrombocytopenia was defined as a platelet count of less than 100,000 per cubic millimeter. In women who had normal blood pressure but proteinuria at baseline, the diagnosis of preeclampsia required thrombocytopenia, or a serum aspartate aminotransferase concentration of ≥ 70 U per liter, or hypertension and either severe headaches, epigastric pain, or worsening proteinuria (either five times the baseline value or twice baseline if the baseline value exceeded 5 g per 24 hours). In women who had hypertension but no proteinuria at baseline, a diagnosis of preeclampsia required the development of proteinuria or thrombocytopenia. A woman was deemed to have preeclampsia if she had an eclamptic convulsion or the HELLP syndrome, defined as hemolysis (serum total bilirubin concentration of ≥1.2 mg per deciliter (20 mmol per liter), a serum lactate dehydrogenase concentration of ≥ 600 U per liter, or hemolytic anemia as determined by the presence of schistocytes on a peripheral smear), elevated serum concentration of aspartate aminotransferase (≥ 70 U per liter), and thrombocytopenia. The records of all the women with apparent preeclampsia, worsening hypertension, new-onset proteinuria, or proteinuria at baseline (≥ 1+) were reviewed independently by three physicians who had to agree unanimously on the validity of the designated outcomes, a policy designed to ensure diagnostic consistency.

The clinical onset of preeclampsia was defined as the time at which a patient met the criteria for preeclampsia as described above rather than the time at which any single symptom first occurred.

### Laboratory methods

Serum samples were collected and aliquoted as part of the Maternal-Fetal Medicine Units Network multicenter randomized controlled trial of low-dose aspirin for the prevention of preeclampsia in high-risk women [4]. Samples were collected between June 1992 and December 1995, aliquoted, frozen and stored at −80°C. Samples were thawed one time (74%) or thawed two times (26%), and the storage time and handling of samples was similar to that of other similar studies including the CPEP cohort. Serum samples were assayed concurrently for sFlt1, sEng and PlGF using commercially available immunoassay kits purchased from R&D Systems (Minneapolis, MN). All kits utilized were from the same manufacturing lot for each specific analyte, a policy designed to minimize variability. ELISAs were validated by performing dilutional parallelism and spike-recovery tests. Correlations between the degree of sample dilution and measured analytes were linear (r^2^ >0.99, all). Calculated recoveries of excess analytes were >94% for all. Measurements of each angiogenic factor were performed in duplicate according to the manufacturer's protocol. All laboratory analyses were performed by personnel unaware of either pregnancy outcome or the high-risk group from which the sera were obtained. In general, samples were diluted 1 to 5 for sFlt1 and sEng, and 1 to 2 for PlGF in order for the samples to fall within the measurable range of the kit's standard curves. A minimal number of samples required re-analysis because the results were outside of the range of the standard curve. Of the total number of serum samples, 135 (5%) required re-assay for sFlt1, 29 (0.9%) for sEng, and 33 (1.2%) for PlGF. The inter-assay variability for each analyte was 10% for sFlt1, 11% for sEng and 7% for PlGF.

### Statistical Methods

Data from all high-risk groups were analyzed separately. Continuous variables were compared using the Wilcoxon rank-sum test and categorical variables using the chi-square test. Change in the concentration of a factor was calculated by taking the difference of the value at study entry and the value obtained between 24 and 28 weeks and dividing by the number of weeks between the two sample collections. Logistic regression was used to calculate odds ratios and the multivariable analysis included gestational age at sample collection, smoking, maternal age, race or ethnic group, body mass index and study treatment group (i.e., aspirin or placebo).

Data were also analyzed cross-sectionally, according to intervals of gestational age and the number of weeks before the onset of preeclampsia. In the intervals, when more than one sample existed per woman, the earliest sample was used. All available samples were used in the gestational age analysis while a matched (1∶1) pair analysis was performed to evaluate differences in relation to the number of weeks before the onset of preeclampsia. Specifically, in the paired comparisons all available samples from subjects who developed preeclampsia were distributed into time blocks according to when the sample was collected in relation to the clinical onset of preeclampsia for each subject (onset of clinically recognized preeclampsia is time 0). Then a separate sample from a control subject from the same high-risk group was matched within one week of the same gestational age in weeks and by treatment group to the sample from the preeclampsia subject. Data were log transformed and the difference was evaluated using the Wilcoxon Signed Rank test.

All P values are two-tailed and a value less than 0.05 was considered statistically significant. No adjustments were made for multiple comparisons. Analyses were performed with the use of the SAS software.

## Results

### Characteristics of the Study Subjects

A total of 2614 serum samples were available to assay. Eight women did not have a study entry sample available for analysis and their fifteen subsequent samples were excluded. Therefore, a total of 2,599 samples were available for this analysis (diabetes, n = 524; chronic hypertension, n = 827; multifetal gestation, n = 574; and previous preeclampsia, n =  674) from 993 women (diabetes, n = 194; chronic hypertension, n = 313; multifetal gestation, n = 234; and previous preeclampsia, n = 252). The original trial included 2,539 women so this analysis represents 39% of the women originally studied. Within each of the high-risk groups, women in this analysis (i.e. women with samples) were similar to women not included (i.e. without samples) for the following characteristics: primigravida, smoking, maternal age and body mass index. There were fewer African American and more Caucasian in the diabetes group and fewer other and more Caucasian in the multifetal group compared to women without a sample. The gestational age at study entry was also slightly earlier in women with samples in the multifetal gestation group compared to women without a sample. Lastly, blood pressure at study entry was slightly lower in women with chronic hypertension and slightly higher in multifetal gestation compared to women without a sample (data not shown).

The characteristics of the 993 study subjects are shown in Table 1. The incidence of preeclampsia in the high-risk groups was 46 of 194 (23.7%) for subjects with diabetes, 78 of 313 (24.9%) for subjects with chronic hypertension, 39 of 234 (16.7%) for subjects with multifetal gestations, and 50 of 252 (19.8%) for subjects with previous preeclampsia. Within this cohort, only 40 subjects (4.0%) were identified as having the onset of preeclampsia before 34 weeks' gestation. The number of samples available within each of the high-risk groups was too few to analyze by early onset of preeclampsia (3 of the 4 risk groups had n<7).

**Table 1:** Study Subject Information According to High-Risk Group.

	Diabetes (n = 194)		Chronic Hypertension (n = 313)		Multifetal Gestation (n = 234)		Previous Preeclampsia (n = 252)	
*Characteristic*	Control (n = 148)	Preeclampsia (n = 46)	Control (n = 235)	Preeclampsia (n = 78)	Control (n = 195)	Preeclampsia (n = 39)	Control (n = 202)	Preeclampsia (n = 50)
Maternal age (years)	26±6	26±6	29±7	30±6	25±6	26±7	24±5	25±6
Body-mass index (kg/m^2^)	28±7	27±8	33±9	34±9	26±7	29±8	28±8	30±9
Gestational ageat study entry (weeks)	17±3	19±4	19±4	19±4	21±3	20±4	20±4	19±4
Primigravida, no. (%)	46 (31)	20 (43)	49 (21)	16 (21)	53 (27)	19 (49)*	---	---
Race or ethnic group, no. (%)		*						
Black	42 (28)	19 (41)	148 (63)	49 (63)	92 (47)	22 (56)	153 (76)	37 (74)
White	91 (61)	27 (59)	65 (28)	18 (23)	78 (40)	14 (36)	42 (21)	13 (26)
Hispanic/Other	15 (10)	0 (0)	22 (9)	11 (14)	25 (13)	3 (8)	7 (3)	0 (0)
Average blood pressure at entry (mmHg)	112±14/67±10	118±15*/70±12	126±15/76±12	132±13*/81±11*	111±11/64±9	116±9*/67±9*	110±11/65±10	115±12*/70±9*
Smoked during pregnancy,no. (%)	34 (23)	5 (11)	40 (17)	13 (17)	28 (14)	3 (8)	31 (15)	9 (18)
Aspirin treatment,no. (%)	78 (53)	18 (39)	112 (48)	44 (56)	101 (52)	14 (36)	102 (50)	23 (46)
Gestational age at delivery (weeks)	37±2	36±2	38±2	36±3*	35±3	35±2	38±2	37±2*
Gestational age at delivery <37 weeks	46 (31)	23 (50)*	46 (20)	35 (45)*	122 (63)	28 (72)	30 (15)	16 (32)*
Onset of preeclampsia before 37 weeks' gestation, no. (%)	---	30 (65)	---	52 (67)	---	27 (69)	---	19 (38)
Infant birth weight (grams)	3288±782	3162±943	3120±689	2797±907*	2334±607	2461±458	3189±642	3135±729

Data are mean ± standard deviation or N (%).

*: p<0.05 compared to group appropriate control.

Overall, there was no difference in maternal age, body mass index, or gestational age at study entry between the preeclamptic subjects within each high-risk group and their representative controls. The preeclamptic subjects in each high-risk group exhibited a modest but statistically significant higher average blood pressure at study entry compared with their representative controls.

The original trial of low-dose aspirin in high-risk patients indicated no effect of aspirin on outcome. In this study, the overall incidence of preeclampsia was not significantly different in the aspirin and placebo treated groups (20% versus 23%, p = 0.31). This was also true in the individual high-risk groups (p≥0.05). In addition, there was no difference in the concentrations of sFlt1, sEng and PlGF between women who were in the aspirin treatment group and those in the placebo group (p≥0.05). Formal tests for interaction between treatment group and the markers were not significant. For these reasons, aspirin treated and placebo groups were combined for these analyses.

### Angiogenic Factors at Study Entry

Subjects provided blood samples at the time of study entry (7–26 weeks' gestation). Samples were obtained from all women before the onset of clinical signs of preeclampsia. The concentration of sFlt1, sEng and PlGF were all statistically significantly higher at study entry in the women with multifetal gestation compared with subjects with pre-existing diabetes, chronic hypertension and previous preeclampsia (p<0.0001 for all, data not shown). In addition, the concentration of PlGF was significantly lower in the diabetic subjects compared with PlGF in the chronic hypertensive and previous preeclampsia high-risk groups (p<0.001, data not shown).

We investigated whether differences in the angiogenic factors at study entry were associated with the subsequent development of preeclampsia. The odds for developing preeclampsia were significantly increased 2 to 3 fold for each 2-fold elevation in the concentration of sFlt1, sEng and the ratio of combined angiogenic factors (sFlt1+ sEng/PlGF) at study entry among women with multifetal gestations who later developed preeclampsia (Table 2). In addition, the odds of developing preeclampsia were significantly decreased by half for each 2-fold elevation in maternal circulating PlGF at study entry among women with multifetal gestations and diabetes (Table 2). The odds of developing preeclampsia was significantly increased by 60% for each 2-fold elevation in circulating sEng at study entry among subjects with chronic hypertension, and for each 2-fold elevation in the angiogenic factor ratio among subjects with diabetes (Table 2). Conversely, none of the factors analyzed or their ratio at study entry were associated with an increased odds of developing preeclampsia among the subjects with previous preeclampsia.

**Table 2:** Odds Ratios for Preeclampsia According to Circulating Angiogenic Factors Measured in Samples Collected at Study Entry.*

Diabetes				
	Controls(N = 148)	Preeclampsia(N = 46)	Unadjusted OR[95% CI]	Adjusted OR[95% CI] †
sFlt1 (ng/ml)	3.60±2.02	3.75±2.33	0.98 [0.64,1.51]	0.98 [0.63,1.53]
sEng (ng/ml)	5.31±1.58	5.75±2.29	1.62 [0.74,3.54]	1.66 [0.70,3.92]
PIGF (pg/ml)	151.14±144.55	156.74±147.51	1.02 [0.78,1.32]	0.59 [0.36,0.95]
sFlt1 + sEng/PIGF	124.94±135.34	124.36±111.64	1.03 [0.80,1.32]	1.61 [1.07,2.48]

Chronic Hypertension				
	Controls(N = 235)	Preeclampsia(N = 78)	Unadjusted OR[95% CI]	Adjusted OR[95% CI] †
sFlt1 (ng/ml)	3.68±3.66	3.83±2.91	1.09 [0.79,1.49]	1.16 [0.83,1.63]
sEng (ng/ml)	5.58±5.73	6.05±3.60	1.51 [0.96,2.39]	1.60 [1.01,2.57]
PIGF (pg/ml)	222.54±236.67	191.61±154.19	0.92 [0.74,1.13]	0.96 [0.71,1.29]
sFlt1 + sEng/PIGF	96.65±214.75	123.71±216.91	1.13 [0.94,1.36]	1.13 [0.90,1.41]

Multifetal Gestation				
	Controls(N = 195)	Preeclampsia(N = 39)	Unadjusted OR[95% CI]	Adjusted OR[95% CI] †
sFlt1 (ng/ml)	5.95±2.47	7.33±5.42	1.55 [0.93,2.68]	2.07 [1.15,3.89]
sEng (ng/ml)	7.04±2.70	8.54±4.50	2.14 [1.14,4.05]	2.98 [1.44,6.36]
PIGF (pg/ml)	554.73±388.10	386.10±318.17	0.61 [0.44,0.82]	0.50 [0.30,0.82]
sFlt1 + sEng/PIGF	40.37±43.20	75.31±67.62	1.80 [1.35,2.44]	2.18 [1.46,3.32]

Previous Preeclampsia				
	Controls(N = 202)	Preeclampsia(N = 50)	Unadjusted OR[95% CI]	Adjusted OR[95% CI] †
sFlt1 (ng/ml)	3.32±1.84	3.16±1.72	0.86 [0.60,1.32]	0.96 [0.61,1.52]
sEng (ng/ml)	5.52±4.93	5.60±2.71	1.25 [0.72,2.12]	1.41 [0.80,2.46]
PIGF (pg/ml)	231.58±218.94	191.35±159.20	0.89 [0.72,1.11]	0.93 [0.67,1.36]
sFlt1 + sEng/PIGF	100.70±207.02	122.37±186.63	1.13 [0.92,1.38]	1.15 [0.83,1.56]

*Data are presented as mean ± standard deviation. OR denotes odds ratio, CI confidence interval. Odds ratios represent the odds of developing preeclampsia for a two-fold increase of the factor among preeclamptic subjects compared to controls.

† Odds ratios were adjusted for gestational age at sample collection, smoking, maternal age, race or ethnic group, body mass index and treatment group.

### Concentration of Angiogenic Factors Across Gestation

We next investigated whether the change in each factor between the sample collected at study entry and the sample collected at visit 2 (between 24 and 28 weeks' gestation) identified women at an increased risk of later developing preeclampsia. A total of 790 of the 993 women had samples collected during these two time points. The odds of developing preeclampsia were significantly increased for each 1-unit increase in the change in circulating sFlt1 among the multifetal gestation and previous preeclampsia high-risk groups (Table 3). In addition, the odds of developing preeclampsia were significantly increased for each 1-unit increase in the change in sEng among the diabetes and multifetal gestation high-risk groups. Finally, the odds of developing preeclampsia were significantly increased for each 1-unit increase in the change in the angiogenic factor ratio among the diabetes and multifetal high-risk groups (Table 3). While these odds ratios are statistically significant, a 1-unit difference in these factors represents a sizable change as evidenced by the values reported in Table 3.

**Table 3:** Odds Ratios for Preeclampsia According to the Change per Week in Circulating Angiogenic Factors between Study Entry and Second Study visit.*

Diabetes			
	Controls(N = 130)	Preeclampsia(N = 37)	Adjusted OR[95% CI] †
Change in sFlt1 (ng/ml)	0.04±0.17	0.07±0.57	1.14 [0.34, 4.84]
Change in sEng (ng/ml)	0.08±0.23	0.27±0.44	4.58 [1.31, 17.5]
Change in PIGF (pg/ml)	33.88±38.92	33.09±57.49	0.99 [0.90, 1.08]
Change in sFlt1 + sEng/PIGF	−8.39±12.21	0.33±25.01	1.10 [1.04, 1.19]

Chronic Hypertension			
	Controls (N = 187)	Preeclampsia (N = 62)	Adjusted OR [95% CI] †
Change in sFlt1 (ng/ml)	0.07±1.04	0.25±0.49	1.62 [0.94, 3.02]
Change in sEng (ng/ml)	0.70±7.01	0.49±1.24	0.99 [0.84, 1.05]
Change in PIGF (pg/ml)	32.88±63.28	28.97±43.72	0.98 [0.90, 1.04]
Change in sFlt1 + sEng/PIGF	−0.54±68.50	12.76±65.88	1.01 [0.99, 1.02]

Multifetal Gestation			
	Controls (N = 154)	Preeclampsia (N = 33)	Adjusted OR [95% CI] †
Change in sFlt1 (ng/ml)	0.15±0.49	0.48±0.81	4.91 [1.95, 16.1]
Change in sEng (ng/ml)	0.27±0.59	0.75±1.39	2.82 [1.51, 5.77]
Change in PIGF (pg/ml)	54.45±114.54	24.15±77.87	0.95 [0.90, 1.00]
Change in sFlt1 + sEng/PIGF	−1.23±6.90	5.52±20.29	1.10 [1.05, 1.17]

Previous Preeclampsia			
	Controls (N = 153)	Preeclampsia (N = 34)	Adjusted OR [95% CI] †
Change in sFlt1 (ng/ml)	0.07±0.28	0.27±1.02	2.58 [1.14, 9.06]
Change in sEng (ng/ml)	0.06±0.54	0.23±1.21	1.48 [0.91, 2.62]
Change in PIGF (pg/ml)	44.18±55.16	33.13±31.13	0.98 [0.87, 1.08]
Change in sFlt1 + sEng/PIGF	−6.70±46.77	−1.99±55.61	1.01 [0.99, 1.02]

*Data are presented as mean ± standard deviation. OR denotes odds ratio, CI confidence interval.

† Odds ratios were adjusted for gestational age and concentration of sample at visit 1, smoking, maternal age, race or ethnic group, body mass index, and treatment group. Odds ratios for sFlt1, sEng and sFlt1+Endo/PIGF represent the odds of developing preeclampsia compared to control subjects for each 1-unit increase of the factor, and the odds ratio for PlGF represents a 10-unit increase of the factor among preeclamptic subjects compared to controls.

**Figure 1:**
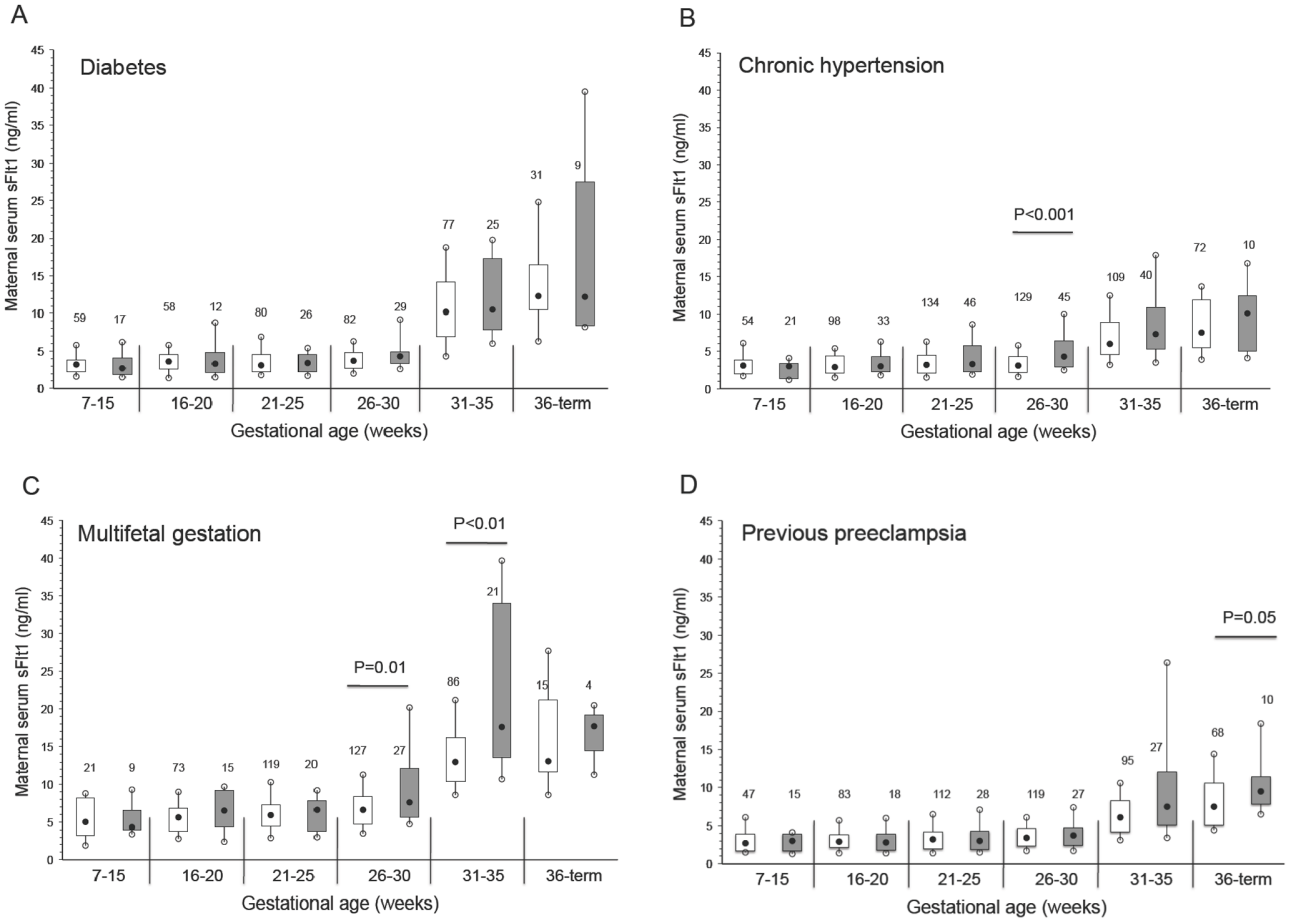
Maternal serum sFlt1 across gestation in women with pre-existing diabetes (A), chronic hypertension (B), multifetal gestation (C), and previous preeclampsia (D). Open boxes are controls and shaded boxes are subjects who developed preeclampsia. The number above each box indicates the number of samples. The filled black circles are the median, the open circles are the 90th and 10th percentiles, and the top and bottom lines of the box are the 75^th^ and 25^th^ percentiles of the data for each group. Statistical significance is indicated by p values.

We also investigated the differences in the maternal concentration of each factor between subjects who developed preeclampsia and their representative controls cross-sectionally within defined intervals of gestational age. In all diagnostic groups, there were modest but statistically significant differences in at least one factor between the subjects who developed preeclampsia and their representative controls at some time in pregnancy. There were no significant differences in sFlt1 between women who developed preeclampsia with pre-existing diabetes compared with control subjects at any stage of gestation (Figure 1A). The concentration of sFlt1 was significantly higher at 26 to 30 weeks' gestation among chronic hypertension (Figure 1B) and multifetal gestation (Figure 1C) subjects who developed preeclampsia. In addition, sFlt1 was significantly higher at 31 to 35 weeks' gestation in multifetal gestation (Figure 1C) subjects who developed preeclampsia. The maternal concentration of sFlt1 tended to be higher at 36 weeks to term among the previous preeclampsia subjects with preeclampsia (p = 0.05; Figure 1D).

**Figure 2:**
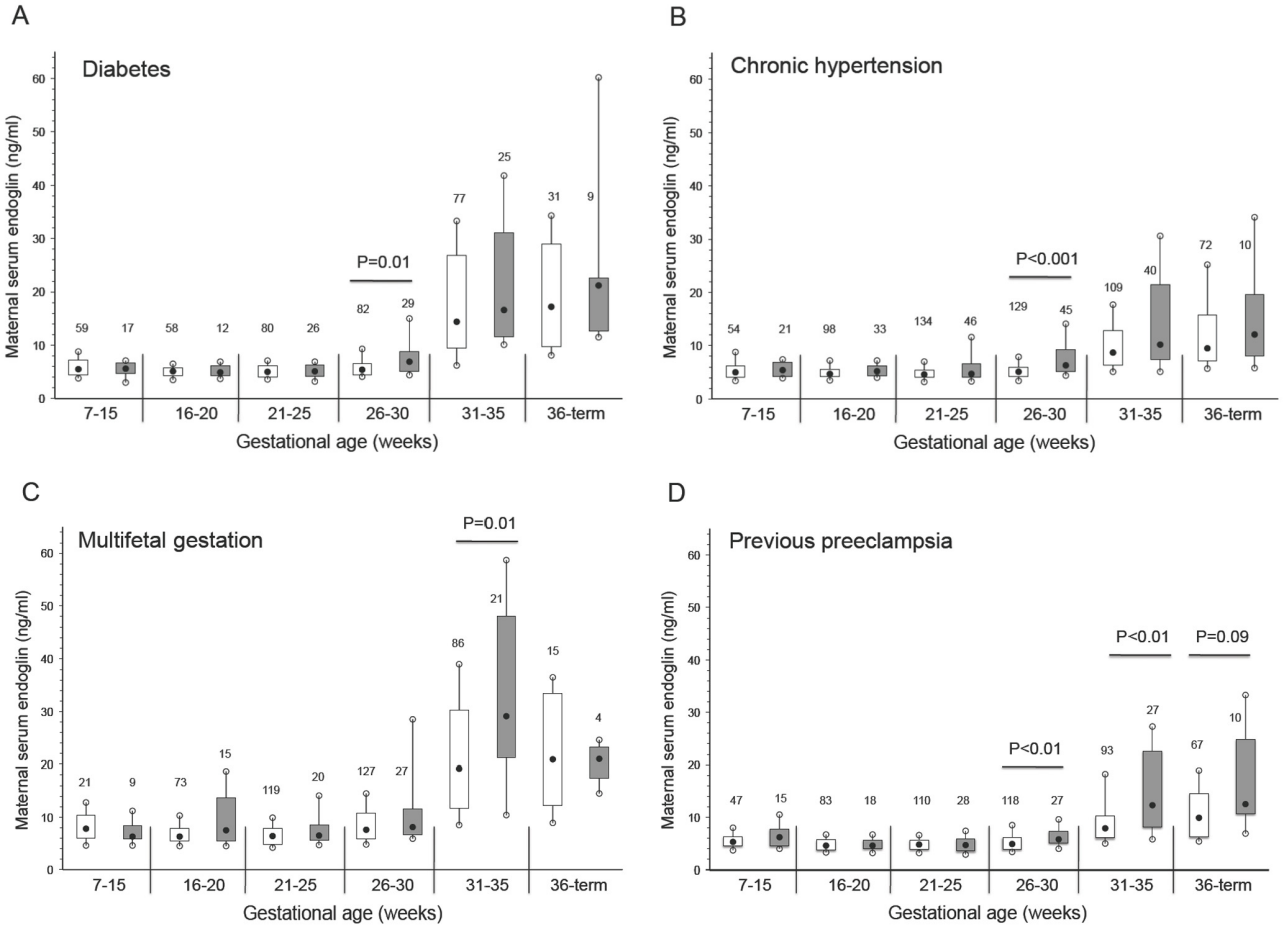
Maternal serum sEng across gestation in women with pre-existing diabetes (A), chronic hypertension (B), multifetal gestation (C), and previous preeclampsia (D). Open boxes are controls and shaded boxes are subjects who developed preeclampsia. The number above each box indicates the number of samples. The filled black circles are the median, the open circles are the 90th and 10th percentiles, and the top and bottom lines of the box are the 75^th^ and 25^th^ percentiles of the data for each group. Statistical significance is indicated by p values.

The concentration of sEng was significantly higher at 26 to 30 weeks in diabetic (Figure 2A), chronic hypertension (Figure 2B) and previous preeclampsia (Figure 2D) subjects who later developed preeclampsia. In addition, sEng was significantly higher at 31 to 35 weeks' gestation in multifetal gestation (Figure 2C) and previous preeclampsia (Figure 2D) subjects who later developed preeclampsia, and sEng tended to be higher at 36 weeks to term among the previous preeclampsia subjects with preeclampsia (p = 0.09; Figure 2D).

**Figure 3:**
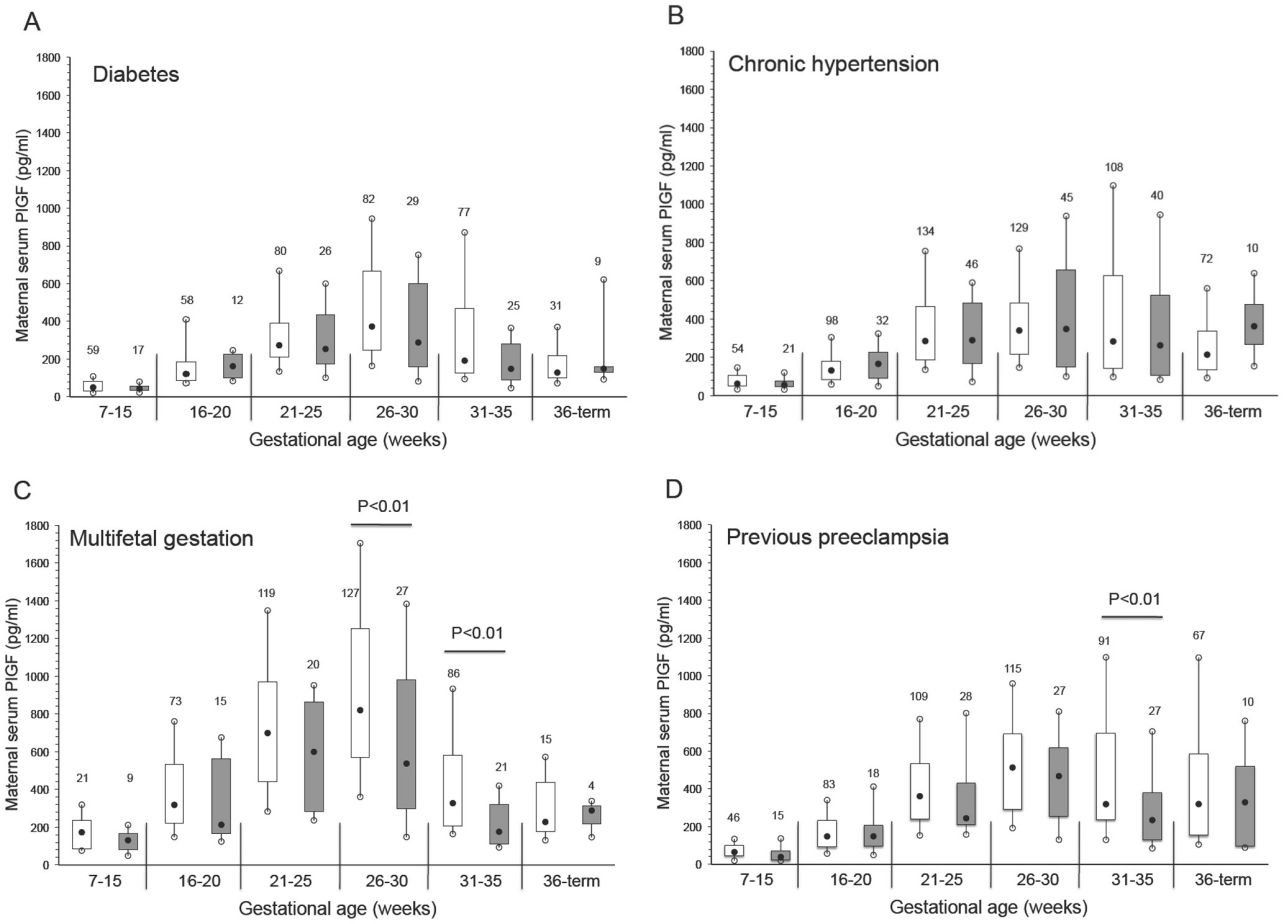
Maternal serum PlGF across gestation in women with pre-existing diabetes (A), chronic hypertension (B), multifetal gestation (C), and previous preeclampsia (D). Open boxes are controls and shaded boxes are subjects who developed preeclampsia. The number above each box indicates the number of samples. The filled black circles are the median, the open circles are the 90th and 10th percentiles, and the top and bottom lines of the box are the 75^th^ and 25^th^ percentiles of the data for each group. Statistical significance is indicated by p values.

The concentration of maternal serum PlGF across gestation is shown in Figure 3. The maternal concentration of PlGF was significantly lower at 31 to 35 weeks' gestation in women with previous preeclampsia and who developed preeclampsia (Figure 3D). Similarly, PlGF was significantly lower at 26 to 30 weeks and 31 to 35 weeks' gestation in multifetal gestation patients with preeclampsia (Figure 3C). Surprisingly, there were no significant differences in PlGF between women who developed preeclampsia compared with control subjects among the women with pre-existing diabetes or chronic hypertension at any stage of gestation (Figures 3A and 3B).

### Concentration of Angiogenic Factors in Relation to the Clinical Onset of Preeclampsia

**Figure 4:**
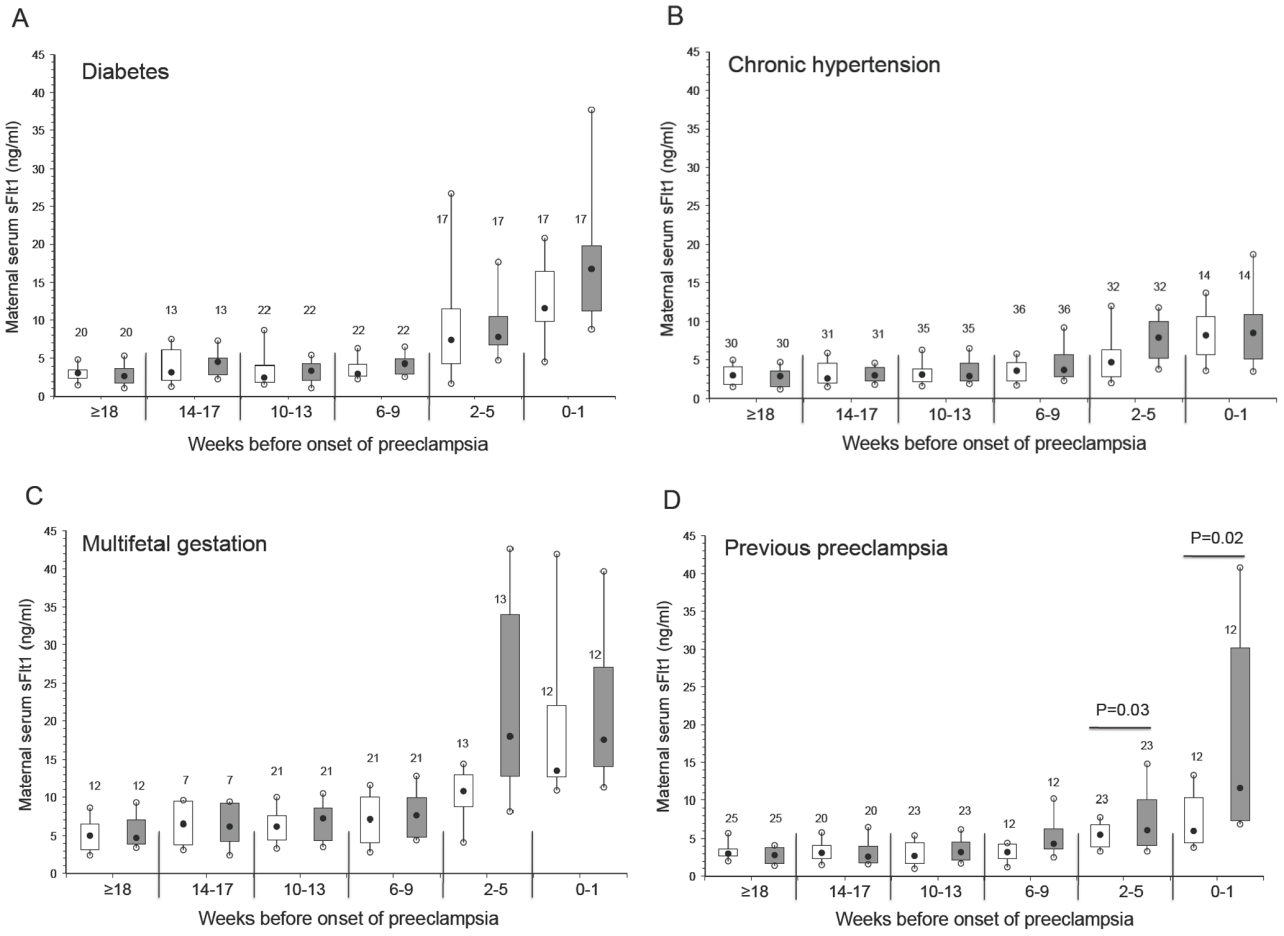
Maternal serum sFlt1 by clinical onset of preeclampsia matched to control samples in women with pre-existing diabetes (A), chronic hypertension (B), multifetal gestation (C), and previous preeclampsia (D). Open boxes are controls and shaded boxes are subjects who developed preeclampsia. The number above each box indicates the number of samples. The filled black circles are the median, the open circles are the 90th and 10th percentiles, and the top and bottom lines of the box are the 75^th^ and 25^th^ percentiles of the data for each group. Statistical significance is indicated by p values.

**Figure 5:**
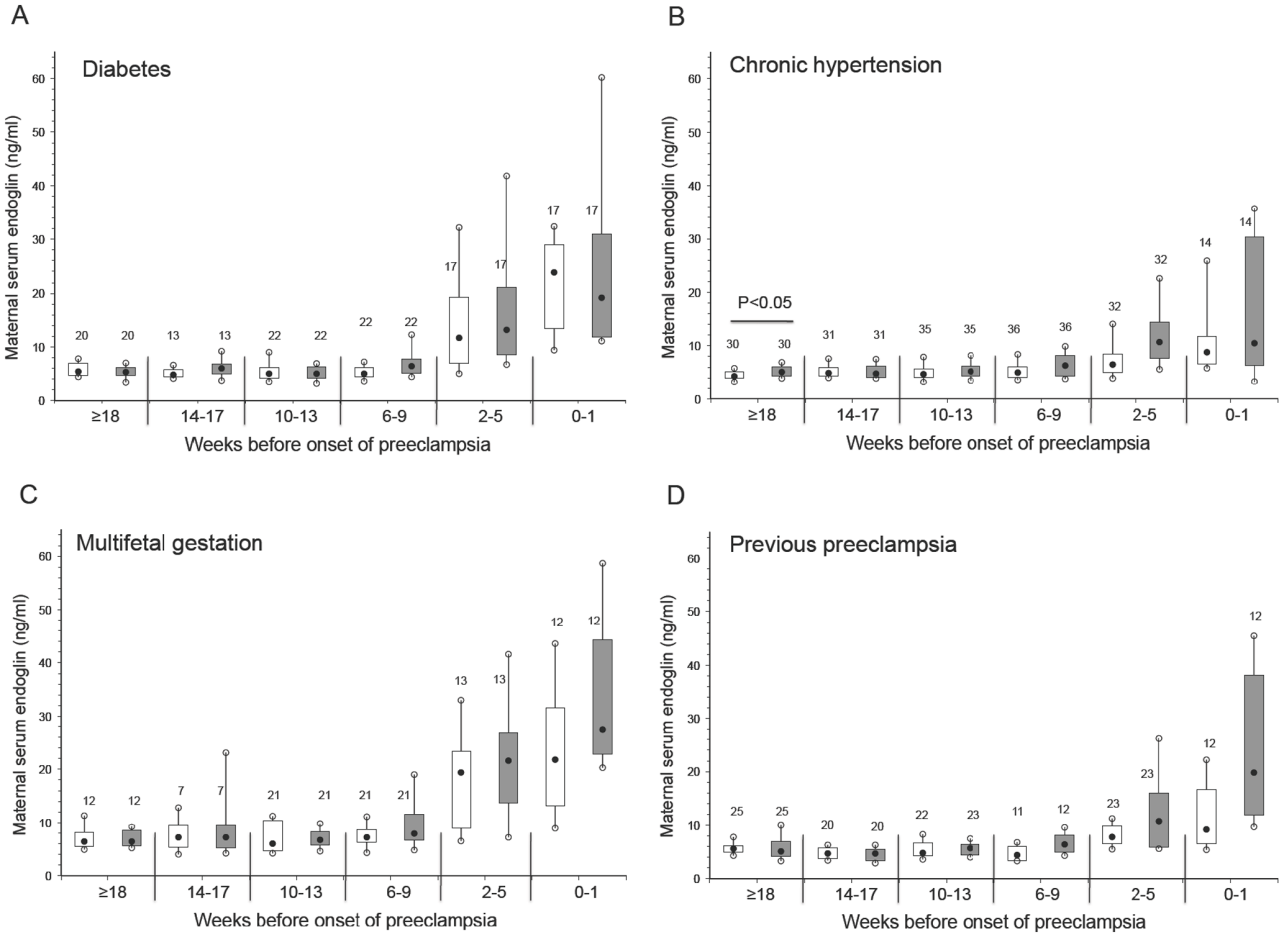
Maternal serum sEng by clinical onset of preeclampsia matched to control samples in women with pre-existing diabetes (A), chronic hypertension (B), multifetal gestation (C), and previous preeclampsia (D). Open boxes are controls and shaded boxes are subjects who developed preeclampsia. The number above each box indicates the number of samples. The filled black circles are the median, the open circles are the 90th and 10th percentiles, and the top and bottom lines of the box are the 75^th^ and 25^th^ percentiles of the data for each group. Statistical significance is indicated by p values.

**Figure 6:**
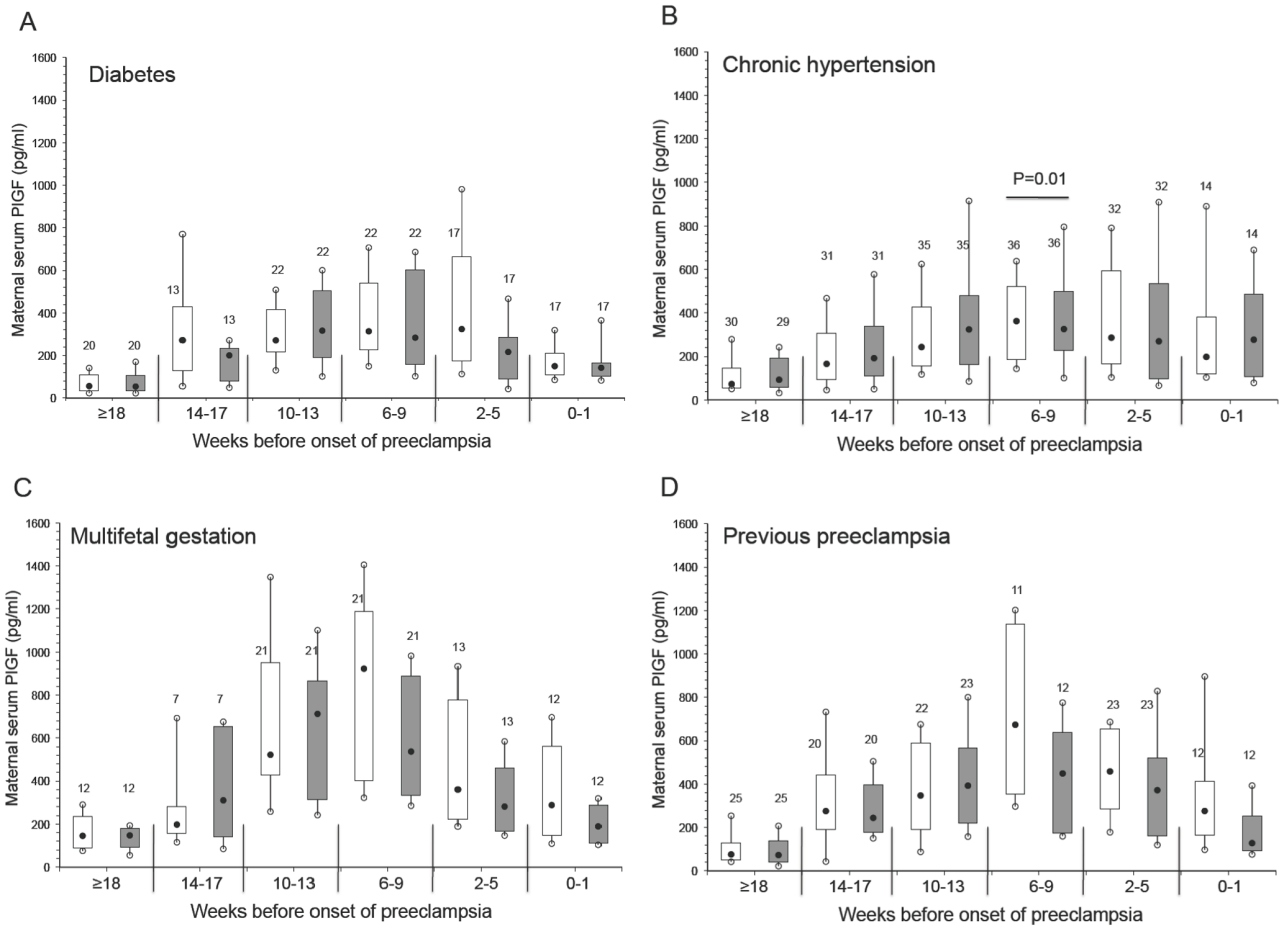
Maternal serum PlGF by clinical onset of preeclampsia matched to control samples in women with pre-existing diabetes (A), chronic hypertension (B), multifetal gestation (C), and previous preeclampsia (D). Open boxes are controls and shaded boxes are subjects who developed preeclampsia. The number above each box indicates the number of samples. The filled black circles are the median, the open circles are the 90th and 10th percentiles, and the top and bottom lines of the box are the 75^th^ and 25^th^ percentiles of the data for each group. Statistical significance is indicated by p values.

We next investigated whether the maternal concentration of the angiogenic factors was different between subjects who developed preeclampsia compared with controls among the four high-risk groups in relation to the clinical onset of the syndrome. Box and whisker plots of matched samples according to the clinical onset of preeclampsia (time 0) for each separate high-risk group are shown in Figures 4 (sFlt1), Figures 5 (sEng) and Figures 6 (PlGF).

Maternal sFlt1 was significantly elevated 2 to 5 weeks before the onset of preeclampsia in the subjects with previous preeclampsia, as well as within 1 week of the clinical onset of preeclampsia (Figure 4D). However, sFlt1 concentrations were not significantly elevated before the onset of preeclampsia in any of the other high-risk groups (Figures 4A, B and C). Similarly, the concentration of maternal sEng was not significantly elevated in any of the high-risk groups immediately before or close to the onset of preeclampsia compared with samples from gestational age matched controls (Figures 5A, B, C and D). Of note, there was a modest but statistically significant elevation in sEng at ≥18 weeks before the onset of preeclampsia among the chronic hypertension patients compared with their matched controls. Lastly, the concentration of maternal PlGF was not significantly lower among any of the high-risk groups (Figures 6A, B, C and D) except at 6–9 weeks in the chronic hypertensive group (Figure 6B). In addition, the ratio of the angiogenic factors was not different in any of the high-risk groups before the onset of preeclampsia compared with samples from gestational age matched controls (data not shown).

## Discussion

There has been considerable interest in the role and possible predictive value of the circulating angiogenic factors sFlt1, sEng and PlGF for preeclampsia as evidenced by a growing literature since 2003. Most studies have focused on low-risk populations while very few have evaluated the utility of these factors in high-risk pregnant populations, and the latter reports are limited in size or have evaluated subjects with different reasons for being classified high-risk together [7], [8], [9], [10], [11], [12]. In our study, maternal circulating sFlt1, sEng and PlGF were significantly higher at study entry in subjects with multifetal gestations compared with other high-risk groups, and PlGF was significantly lower in the diabetic subjects. The odds of developing preeclampsia were significantly increased for each 2-fold elevation in sFlt1, sEng and the angiogenic factor ratio, and were significantly decreased for each 2-fold elevation in PlGF at study entry in subjects with multifetal gestations. Similarly, the odds of developing preeclampsia are significantly decreased by half for each 2-fold elevation in maternal PlGF among diabetics, and increased by 60% for each 2-fold elevation in sEng and the angiogenic factor ratio at study entry for subjects with chronic hypertension and diabetes respectively. While these odds ratios are statistically significant, the confidence intervals associated with them are wide, and the magnitude of the elevation in the angiogenic factors in early pregnancy is large, which does not lend confidence to the possibility of these factors individually as useful predictors of preeclampsia in these high-risk groups. However, these data do not preclude the possible utility of these factors in a multiple marker screen for the prediction of preeclampsia.

We also observed that the change in sFlt1 and sEng, and the change in the ratio of the angiogenic factors between study entry (7–26 weeks' gestation) and the second study visit (24–28 weeks' gestation) was associated with a significant increase in the odds of developing preeclampsia among these high-risk populations. These data are similar to that previously reported among low-risk patients, such that women who developed preeclampsia were more likely to show greater sequential changes in sFlt1 and sEng in early pregnancy, and these changes were most pronounced in preterm preeclampsia [13]. These data suggest that perhaps the absolute concentration of these antiangiogenic factors is not as important as the relative change in these factors within an individual in early pregnancy, and that similar pathophysiologic mechanisms may be at work in low-risk and high-risk populations. While these data are intriguing, the absolute change in these factors associated with an increased risk of preeclampsia is large.

We observed that sFlt1 and sEng were statistically higher, and PlGF was significantly lower at 26 to 30 weeks' gestation or later during pregnancy in subjects who later develop preeclampsia in some but not all of the four high-risk groups. In addition, we observed that there were relatively few significant differences in the concentration of these angiogenic factors that precede the onset of the clinical syndrome of preeclampsia. In most cases, however, even when the differences are not significant, the relationship of mean values of these angiogenic factors is similar to that observed in low-risk women with higher sFlt1 and sEng and lower PlGF in women who later develop preeclampsia suggesting these factors may play a role in the pathophysiology of preeclampsia in these high-risk groups. Nonetheless, the small differences in these factors or their ratio make it unlikely they will be clinically useful as individual predictive markers of preeclampsia in these high-risk groups.

Among low-risk pregnant patients, sFlt1 has been reported to be significantly elevated by 33 to 36 weeks' gestation and >5 weeks before the clinical onset of preeclampsia [10]. Similarly, maternal concentrations of sEng are significantly elevated at 25 to 28 weeks' gestation and 9 to 11 weeks before clinical onset of preterm preeclampsia, and 33 to 36 weeks' gestation and 9 to 11 weeks before the clinical onset of term preeclampsia [9]. Maternal concentrations of PlGF are also reported to be significantly lower by 13 to 20 weeks' gestation among women who later develop preeclampsia compared with similar low-risk control subjects [10]. In contrast to low-risk subjects, there has been limited investigation of the differences in these angiogenic factors across pregnancy among high-risk subjects. Moore Simas et al. evaluated the predictive value of maternal sFlt1 and PlGF between 22 and 36 weeks' gestation in a combination of several high-risk populations including patients with chronic hypertension and pre-gestational diabetes [14]. This study reported that mean serum sFlt1 and sFlt1/PlGF ratio were higher at 22 weeks' gestation in subjects who developed early onset preeclampsia (<34 weeks) compared with uncomplicated controls, and that serum sFlt1 was significantly higher after 31 weeks' gestation in subjects with late onset preeclampsia (>34 weeks). The authors conclude that these angiogenic factors may be predictive of preeclampsia in high-risk populations. However, this study is limited by a small sample size, with 12 preeclamptic subjects total, 5 with early onset and 7 with late onset preeclampsia; as well as a significantly mixed population of high-risk groups (all 5 early onset preeclamptic subjects had chronic hypertension, 2 with pre-gestational diabetes, 1 with renal disease, and 2 with previous preeclampsia) making it difficult to conclude the significance of these factors within any single high-risk group. A study by the same group also reported that maternal serum sFlt1 and PlGF are significantly elevated in subjects with multifetal gestations as early as 22 weeks' gestation [15]. These data are similar to the data reported in this study, as well as our finding that sEng is also significantly higher in all pregnant women with multifetal gestations. The explanation for the elevated angiogenic factors in women with multifetal gestations is likely related to the increased placental mass, however we lack sufficient information to provide a complete explanation. This same explanation was also proposed by Maynard et al. as well as reporting a significant correlation between circulating sFlt1 and placental mass in multifetal gestations (r = 0.62, p = 0.0002) [15]. In addition, a recent study by Sibai et al. investigated the predictive value of maternal sFlt1 and PlGF between 12 to 19 weeks and 24 to 28 weeks' gestation among 704 patients with previous preeclampsia and/or chronic hypertension (14.7% had both conditions) [16]. The authors reported that PlGF concentrations were significantly lower at baseline and sFlt1 concentrations were higher at 24 to 28 weeks' gestation in subjects who later developed preeclampsia, and these differences were most noticeable among subjects who developed preeclampsia before 27 weeks' gestation. However, overall these differences were modest, and the sensitivities and positive predictive values were low suggesting that the clinical utility of these markers is limited.

In general, the patterns of angiogenic factor concentrations across pregnancy in this study (i.e. elevated sFlt1 and sEng and low PlGF among subjects who develop preeclampsia) are similar to those presented in studies of low-risk pregnant women. However, differences in these angiogenic factors between high-risk women who develop preeclampsia compared with high-risk women who do not develop preeclampsia appear more modest, similar to the data presented by Sibai et al [16]. What then might be the possible reasons for these differences between studies involving low-risk pregnant women compared with high-risk pregnant women? One notable technical difference is a change in the formulation of the commercial kit for the measurement of sFlt1. Most prior studies and our study used the ELISA kit from the same company (R&D Systems; Minneapolis, MN). A formulation change in this kit made since the publication of several of the prior studies resulted in higher measured concentrations of sFlt1 than reported previously among low-risk pregnant women. However, the formulation of the commercial ELISA kits for sEng and PlGF have not changed, and the overall pattern of maternal serum sFlt1 concentrations in high-risk women is similar to that of low-risk women.

In this study we decided a priori to analyze these high-risk groups separately. Preeclampsia is a heterogeneous condition and it is quite likely that the path to preeclampsia might be different between these disparate high-risk conditions. Despite this, limitations in sample size are an unlikely explanation since the number of high-risk subjects within each group in this study is considerably greater than that presented in other studies of high-risk subjects, and the number of samples in this study was in many cases as numerous as those used in studies of low-risk women. However, despite the large number of subjects and samples in this study, we were not able to separate groups according to preterm severity criteria (<34 weeks' gestation) which is when the most pronounced differences in these angiogenic factors appear among low-risk subjects, because of an insufficient number of these preterm subjects in each of the high risk groups [9]. An additional limitation of this study is the fact that the original parent study was not designed to predict preeclampsia. However, despite these limitations, all samples in this study were analyzed in a blinded fashion, the inter-assay variability for the measurement of each angiogenic factor was within acceptable limits and similar to that presented in other studies, the overall variability of the data particularly for sEng was low and similar to that presented in other studies, the majority of samples were thawed only one time (74%) or thawed two times (26%), and the storage time and handling of samples was similar to that of other studies including the CPEP cohort which reported similar values to those reported in this study [13] [17]. Importantly, aspirin was found to have no significant effect on the concentration of any of the angiogenic factors. It is possible that difficulty in determining the clinical diagnosis of preeclampsia in subjects with chronic hypertension and especially these and other subjects with preexisting proteinuria may have affected the results of this study. However, the diagnosis of preeclampsia in these subjects was critically evaluated by chart reviews in the initial parent study. In addition, this limitation provides additional rationale for maintaining separate diagnostic groups. The clinical diagnosis of preeclampsia in patients with previous preeclampsia or multifetal gestations was not confounded by preexisting conditions, yet even these high-risk groups had only modest differences in the measured angiogenic factors.

In conclusion, we have observed that the maternal concentration of the angiogenic factors sFlt1, sEng and PlGF are significantly higher among women with multifetal gestations compared with other high-risk groups including: pre-existing diabetes, chronic hypertension and previous preeclampsia. The sequential change in sFlt1, sEng and the ratio of these angiogenic factors in early pregnancy is associated with a significant increase in the odds of developing preeclampsia. However, these sequential changes need to be large. Cross-sectional analysis of maternal sFlt1, sEng and PlGF show modest significant differences of at least one of these factors during the third trimester in women who develop preeclampsia in all high-risk groups. Only sFlt1 was significantly higher 2 to 5 weeks before the clinical onset of preeclampsia in women with previous preeclampsia. The significant differences in sFlt1, sEng and PlGF evident in women at high-risk of preeclampsia may support a role for these factors in the pathophysiology of preeclampsia. However, we conclude that the concentration of these circulating angiogenic factors is unlikely to be useful in predicting preeclampsia in these high-risk populations.
